# Structural Response of Polyethylene Foam-Based Sandwich Panels Subjected to Edgewise Compression

**DOI:** 10.3390/ma6104545

**Published:** 2013-10-16

**Authors:** Antonio Boccaccio, Caterina Casavola, Luciano Lamberti, Carmine Pappalettere

**Affiliations:** Dipartimento di Meccanica, Matematica e Management, Politecnico di Bari, Viale Japigia 182, 70126 Bari, Italy; E-Mails: a.boccaccio@poliba.it (A.B.); lamberti@poliba.it (L.L.); c.pappalettere@poliba.it (C.P.)

**Keywords:** foam-based coresandwich panels, edgewise compression, buckling, out-of-plane displacement, projection moiré, nonlinear finite element analysis, facesheet-to-core thickness ratio, facesheet/core interface waviness

## Abstract

This study analyzes the mechanical behavior of low density polyethylene foam core sandwich panels subjected to edgewise compression. In order to monitor panel response to buckling, strains generated in the facesheets and overall out-of-plane deformations are measured with strain gages and projection moiré, respectively. A finite element (FE) model simulating the experimental test is developed. Numerical results are compared with moiré measurements. After having been validated against experimental evidence, the FE model is parameterized, and a trade study is carried out to investigate to what extent the structural response of the panel depends on the sandwich wall construction and facesheet/core interface defects. The projection moiré set-up utilized in this research is able to capture the sudden and very localized buckling phenomena occurring under edgewise compression of foam-based sandwich panels. Results of parametric FE analyses indicate that, if the total thickness of the sandwich wall is fixed, including thicker facesheets in the laminate yields a larger deflection of the panel that becomes more sensitive to buckling. Furthermore, the mechanical response of the foam sandwich panel is found to be rather insensitive to the level of waviness of core-facesheet interfaces.

## 1. Introduction

Sandwich structures made up of low density polyethylene facesheets separated by a lightweight polyethylene foam core represent an excellent solution to many design problems in the automotive field [1]. The mechanical properties of these panels can be improved by properly selecting the manufacturing process, although it is very difficult to establish a direct relationship between manufacturing parameters and mechanical properties. Among manufacturing techniques, rotational molding is an innovative process, which allows one to build the entire sandwich component within just one step, thus improving adhesion and reducing discontinuities between facesheets and the core. Conversely, in traditional techniques, core and skins are manufactured separately and then bonded in a successive step. Therefore, rotational molding allows manufacturing time to be shortened, as well as mechanical characteristics of the panel to be improved [2].

Extensive research was carried out on mechanical characterization of foam-based sandwich structures and assessment of their structural response under specific loading/testing conditions, such as, for example, through-thickness compression [3,4] and edgewise compression [5], indentation [6,7] and low/high velocity impact tests [8,9,10]. Other authors studied the effects on the strength of damages included in the foam or at the skin-core interface [11,12,13], as well as the crack growth process in sandwich panels under fatigue loading [14,15]. The mechanical behavior of these panels subject to shock loading also was extensively studied [16,17].

Theoretical/numerical models and experimental techniques were developed to study buckling phenomena in sandwich panels under various loading conditions [18,19,20]. Fringe projection techniques [21,22] are naturally suited for monitoring the large out-of-plane displacements that may occur when thin-walled structures, like sandwich panels, experience buckling. The projected lines are modulated by the specimen surface: if lines are projected onto a curved surface, the curvature and spatial frequency of these lines will change; conversely, if lines are projected onto a plane, they remain straight and parallel, but their spacing may change. Fleming* et al.* [23,24] and Burner* et al.* [25] demonstrated that projection moiré could accurately measure displacements of models subject to aerodynamic loads. Featherston and Lester [26] monitored the post-buckling behavior of thin panels with projection moiré. Other examples of experimental buckling analysis of thin-walled stiffened panels are documented in Falzon and Aliabadi [27]. The most recent development in projection moiré is the four-projector setup proposed by Sciammarella* et al.* [28,29,30]. A simplified version of that optical set-up employing only two projectors and one camera is utilized in this research.

In spite of the huge number of studies available in literature on the characterization of the mechanical behavior of foam-based sandwich panels, the mechanisms driving buckling deformations and propagation of buckles through the structure still remain poorly understood. In particular, structural behavior under compression is probably the most critical issue, also because of the lack of official standards regulating the execution of compression tests on foam sandwich panels. In view of this, the main objectives of the present research are the following: (i) to investigate in detail the mechanical response of low density polyethylene foam-based sandwich panels subject to edgewise compression; (ii) to evaluate to what extent buckling response is sensitive to sandwich wall construction (*i.e.*, to the ratio between facesheet thickness and core thickness) and to geometric imperfections included in the laminate, such as the level of waviness of core-facesheet interfaces. For this purpose, projection moiré is utilized to measure the deformed shape and to monitor the progress of buckling in the tested panels. Compression tests are then simulated by a parametric finite element (FE) model, including a variable sandwich wall construction. The FE model is validated against experimental evidence.

## 2. Experimental Tests and Finite Element Analysis

**Figure 1:**
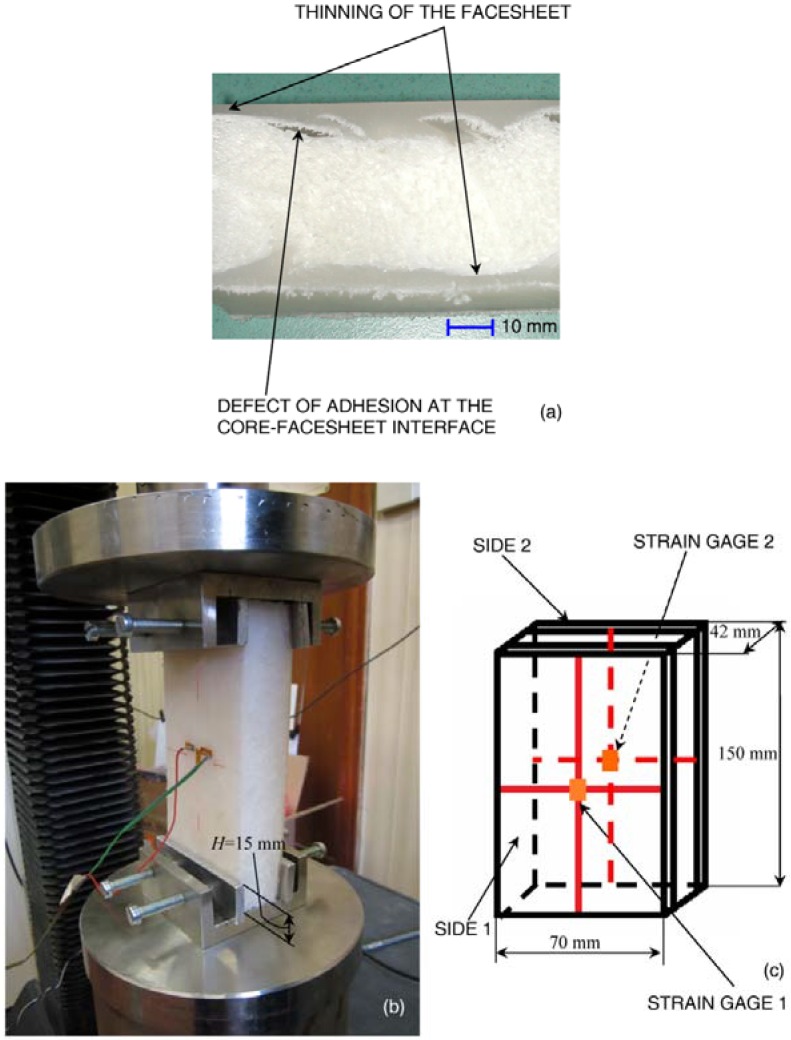
(**a**) Facesheet thinning and core-facesheet adhesion defects; (**b**) loading frame utilized for edgewise compression tests; and (**c**) specimen schematic with indication of strain gage locations.

A set of 10 specimens were cut from low density polyethylene foam-based sandwich panels produced through rotational molding. Each specimen was 150 mm long, 70 mm wide and 42 mm thick (see the schematic in Figure 1c); nominal dimensions were verified for each specimen before carrying out compression tests. In order to have facesheets of equal length for each specimen, the cutting tool was equipped with a guide to keep the cutting plane orthogonal to the external surfaces of facesheets the most as was feasible. While the external surfaces of facesheets are flat, interfaces between the core and the two facesheets may be irregular and present a certain level of waviness, depending on the manufacturing process. Hence, for each of the six faces of the panels, high resolution images were recorded to detect and classify all manufacturing defects near sample edges. Figure 1a shows that there are essentially two types of defects: thinning of facesheets and imperfect adhesion at core-facesheet interfaces.

In edgewise compression tests, the specimen is submitted to in-plane loading in the longitudinal direction. The purpose of this test is two-fold: (i) to analyze the mechanical behavior of facesheets that are much thinner than the core (in the present case, facesheets account for about 10% of the total thickness of the sandwich wall); and (ii) to analyze the contribution of the foam core to the compression strength. Due to the lack of standards specifically concerned with compression tests on foam-based sandwich panels, the experimental tests carried out in this study partially were based on the ASTM C364 standard [31], which, however, deals with generic sandwich structures.

### 2.1. Edgewise Compression Tests and Strain Measurements

Compression tests were conducted with an electromechanical testing machine (MTS Alliance RT/30, MTS Systems, Eden Prairie, MN, USA). Special fixtures, including aluminum plates with a depth of 15 mm, were designed to clamp the specimen into the loading frame (see Figure 1b). The plates are slightly pressed against facesheets in order to prevent lateral displacement without causing pre-deformation of the specimen. The crosshead speed was set equal to 0.5 mm/min; the total end-shortening imposed on the top edge of the panels was 10 mm. Strain gage rosettes with a 3-mm gage length and 120 Ω electrical resistance were bonded onto facesheets, one rosette on each side; the central grid was aligned with the direction of loading. The schematic of Figure 1c reports the nominal dimensions of tested specimens and shows the position of strain gage rosettes on the outer side of facesheets. Loading and end-shortening signals, as well as strain gage output signals were recorded by System 5000 (Vishay Micro Measurements^®^, Raleigh, NC, USA).

### 2.2. Projection Moiré Measurements

The out-of-plane displacement field of the panels was investigated in detail with the multi-projector moiré system developed by Sciammarella* et al.* [28,29,30]. Projection moiré relates out-of-plane deformations with the spatial modulation of a grating projected onto the specimen surface [21,22]. By comparing the line pattern modulated by the deformed object with the line pattern projected on a reference plane, it is possible to obtain moiré fringes that contain the displacement information.

**Figure 2:**
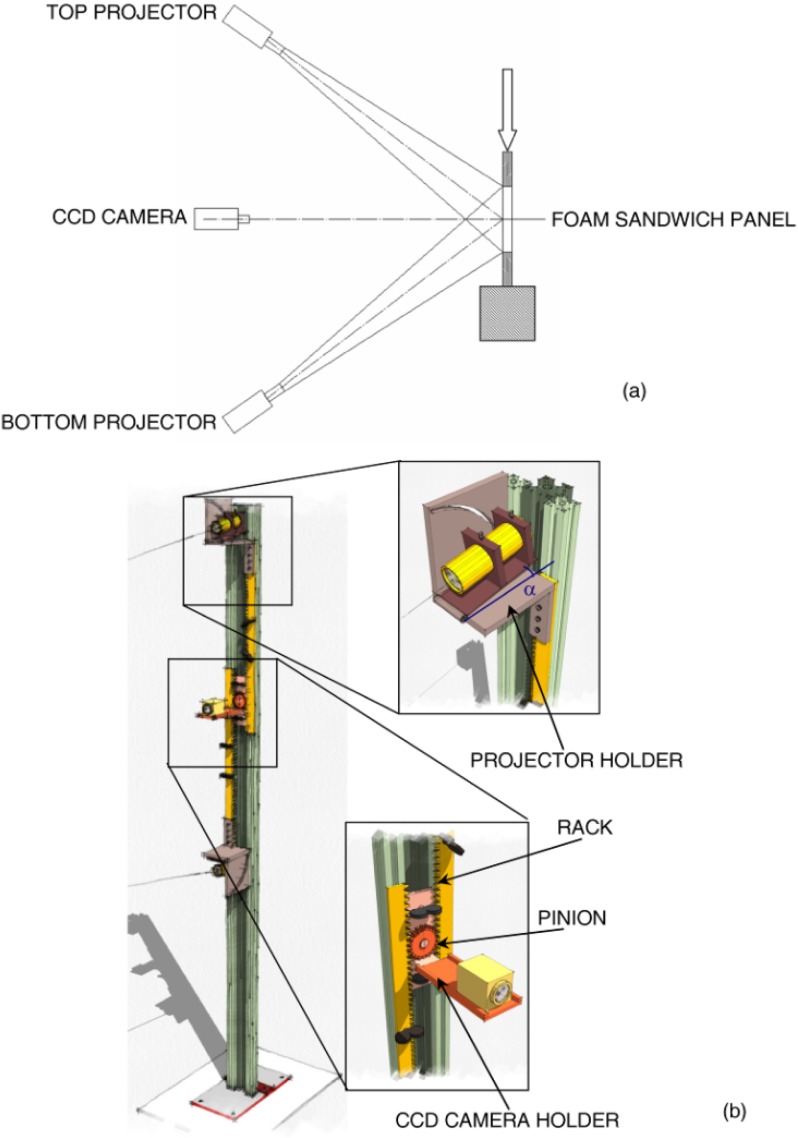
(**a**) Schematic of the two-projector moiré set-up utilized to measure out-of-plane deformation of foam-based sandwich panels; (**b**) the frame supporting the optical set-up.

Since a surface is a tensor entity, the optical set-up should always include four projectors to recover curvature information. If the surface to be measured is prevalently curved with respect to the horizontal direction, as happened in the present case, it is sufficient to have only two projectors (Figure 2a). However, the two projectors must be placed symmetrically with respect to the optical axis of the sensor and project non-collimated structured light onto the sample surface. This allows the ideal condition of projection from infinity to be achieved. Similar to classical Young’s interference occurring between two point sources, a system of lines travelling in the space is generated from the combination of the structured light wavefronts that originate from the two projectors. By changing the illumination angle, θ, made by the optical axis of each projector with the optical axis of the sensor and utilizing gratings of different pitch, it is possible to achieve the required value of sensitivity and contour objects with dimensions ranging from a few microns to meters.

Two different sets of images must be acquired in moiré measurements. The first set includes two images of the grating projected onto the reference plane by the top and bottom projectors, respectively. The second set of images includes the images of the projected grating that has been modulated by the surface of the deformed sample. Reference images are stored in the computer memory as the moiré system is calibrated. Let ϕ_RP-T_(*x*,*y*) and ϕ_RP-B_(*x*,*y*), respectively, be the phases of the projected gratings in the reference plane by the top and bottom projectors. The phase of the equivalent grating projected onto the reference plane is obtained by subtracting the two component phases:

(1) $$\Delta \phi_{\text{RP}}(x,y)=\phi_{\text{RP-T}}(x,y)-\phi_{\text{RP-B}}(x,y)$$

Similarly, if $\phi_{\text{S-T}}(x,y)$ and $\phi_{\text{S-B}}(x,y)$, respectively, are the phases of the deformed grating obtained by illuminating the object with the top and bottom projectors, the total phase of the modulated grating is:

(2) $$\Delta \phi_{\text{S}}(x,y)=\phi_{\text{S-T}}(x,y)-\phi_{\text{S-B}}(x,y)$$

The phase difference, $\Delta \phi_{\text{TOT}}(x,y)$, between deformed and reference configurations is:

(3) $$\Delta \phi_{\text{TOT}}(x,y)=\Delta \phi_{\text{S}}(x,y)-\phi_{\text{RP}}(x,y)$$

The height, *z*(*x,y*), of each point of the surface to be contoured with respect to the reference plane, corresponding to the out-of-plane displacement, is:

(4) $$z\left(x,y\right)=\Delta S\cdot \frac{\Delta \phi_{\mathrm{TOT}}\left(x,y\right)}{2\pi }$$

where the sensitivity, Δ*S*, is equal to *mp*_o_*/*2sinθ; *p*_o_ is the nominal pitch of the projected grating; and *m* is the magnification of the optical set-up. Replacing the expression of Δ*S*, Equation (4) can be rewritten as:

(5) $$z\left(x,y\right)=\frac{mp_{o}}{2\text{sin }\theta }\cdot \frac{\Delta \phi_{\mathrm{TOT}}\left(x,y\right)}{2\pi }$$

Measurements performed on several specimens from the micron to the meter range demonstrate that the accuracy of the two-projector moiré set-up is of the order of some hundredths of the sensitivity of the optical set-up [29,30].

Figure 2b describes in detail the metallic frame designed and realized *ad hoc* for the present research. Three aluminum bars were assembled together to guide the movement of the projectors and CCD camera. A gear engages with two racks that support the projectors. The relative position of projectors and their symmetry with respect to the sensor optical axis are controlled by the gear. The support of each projector is fixed to the frame through holders that can rotate about a horizontal axis. The graduated scale of the holder allows one to line up the optical axis of each projector with the center of the sample. The gear is fixed to a support that includes also a plate carrying the sensor. By properly translating the racks and the gear along the vertical direction, it is possible to perform measurements on panels of different dimensions that may be tested on different machines. In the present experiments, two LTPR3W/R diode projectors (Opto Engineering^®^, Mantova, Italy) carrying objectives of 50° beam aperture and a XCL-5000 5 Mega CCD camera (VisionLinksrl^©^, Seregno, Italy) were utilized.

Before executing optical measurements, the electrical wires of strain gages were positioned so as to minimize any noise affecting the acquired image. Furthermore, the illumination angle, θ, was chosen so as to minimize the shadow cone near the aluminum grips where the out-of-plane displacement field cannot be monitored. A very thin layer of diffusing powder was deposited on the specimens to improve the contrast of the projected lines. The CCD camera sensor plane was disposed parallel to the surface of the sample, and its center was lined up with the center of the sample under investigation.

The surface of the unloaded specimen was taken as the reference plane. This can be done, as the outer facesheets of the specimens are flat. A preliminary calibration procedure was conducted to determine the size of the pixels, Δ*x* and Δ*y*, in *X*-*Y* coordinate directions for the recorded images: it was found that Δ*x* = Δ*y*= 68.97 μm. The nominal pitch of the projected grating *p*_o_ was 127 μm (*i.e.*, 200 lines/in). Because of the large aperture of the beams emerging from projectors, the magnification of the optical set-up was *m* = 37.495. Since the illumination angle was set as θ = 30° (this allowed shadowed areas in the recorded images to be minimized), the resulting sensitivity Δ*S* of the optical set-up is 4762 μm. The illumination angle was precisely determined from the ratio between the nominal pitch *p*_o_ and the value of projected pitch *p*_j_ measured in the reference plane at the intersection between the optical axis of each projector and the optical axis of the camera.

### 2.3. Finite Element Analysis

In order to gather more insight into the structural behavior of the foam-based sandwich panels tested in this research, a FE model simulating the experimental tests was developed. As mentioned before, the external surface of each facesheet is flat and regular, while the interfaces between the core and the two facesheets are irregular. For this reason, the FE model reproduced “point-by-point” the actual thicknesses of facesheets and core.

**Figure 3:**
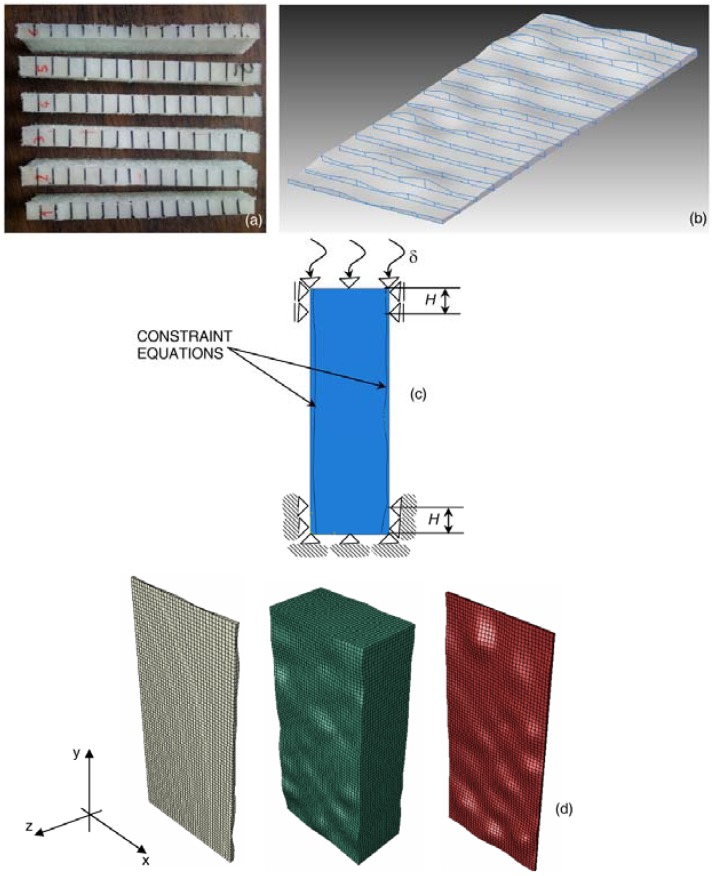
(**a**) Slides cut in the direction parallel to the end-shortening imposed onto the specimen; (**b**) typical 3D reconstruction of facesheets; (**c**) schematic of boundary conditions and loads acting in the compression tests; and (**d**) FE model of the sandwich panel, including different meshes for the core and facesheets.

Six equally spaced slices were cut from specimen 2 in the longitudinal direction, parallel to the loading direction. The thickness of each facesheet was measured at the 16 points that limit the black lines shown in Figure 3a by means of a digital caliper with ±20 μm accuracy. Hence, 16 cross sections were reconstructed for each facesheet slice. The volume occupied by the facesheet was defined by enveloping the reconstructed sections (Figure 3b). The 3D-CAD model of the core was instead defined by subtracting the total volume occupied by the facesheets from the prismatic volume defined by the nominal dimensions of the specimen; these Boolean operations were performed with Solid Edge ST3^®^ (Siemens PLM Software, Plano, TX, USA). The irregularly shaped interfaces between the core and the facesheets are outlined in Figure 3b,c.

Since slices were cut from the specimen after the edgewise compression test, measured values of thickness could actually include residual deformations. For this reason, facesheet thickness was measured at several locations near specimen edges (*i.e.*, where digital caliper could physically access) before executing the compression test. After the test, the same dimensions were again measured, and the difference in thickness was always marginal. Therefore, residual deformation has a very marginal effect on the 3D reconstruction of the specimen and, hence, on the results of FE analysis.

The 3D-CAD models of core and facesheets were imported in the ABAQUS^®^ Version 6.10 finite element program (DassaultSystèmes, Vélizy-Villacoublay, France) and discretized in eight-node hexahedral elements (C3D8) (see Figure 3d). The resulting mesh, obtained via convergence analysis, included 57,750 elements and 68,400 nodes. The compression load acting on the specimen and the boundary conditions realized in the experimental set-up were reproduced in the FE model (see Figure 3c): the nodes of the bottom edge of the model, as well as the nodes located in the outer facesheet regions in contact with the aluminum plates are clamped or can move only in the loading direction, respectively. A vertical displacement of 10 mm corresponding to the total end-shortening given to the panel was imposed on the nodes of the upper side of the FE model and to the outer facesheet nodes located within the region of the FE model limited by the height, *H*, of the aluminum clamping plates. This corresponds to assuming that there is enough friction between plates and facesheets to make them perfectly adhere. However, the real situation was somehow in between adhesion and sliding. In order to evaluate this effect, preliminary FE analyses were run assuming either perfect adhesion (*i.e.*, 10 mm end-shortening imposed on all nodes of the outer facesheets covered by aluminum plates) or full sliding (*i.e.*, end-shortening imposed only on the nodes of the specimen top side). Because of the presence of the clamping plates that prevent lateral displacements regardless of having perfect adhesion or full sliding, the difference between out-of-plane displacements for both cases was found not to be significant. Constraint equations were utilized to connect the nodes of the inner surfaces of the facesheets with the corresponding nodes of the core.

Facesheets were modeled as an elastic-plastic material. Mechanical properties given in the input to the FE model are listed in Table 1. The core was modeled as a compressible hyperelastic foam material. The strain energy function can be expressed in terms of principal stretches, $\bar{\lambda_{1}}$, $\bar{\lambda_{2}}$ and $\bar{\lambda_{3}}$, and volume ratio, *J* [32]:

(6) $$W=\sum_{i=1}^{N}\frac{2\mu_{i}}{{\alpha_{i}}^{2}}\left[{\bar{\lambda_{1}}}^{\alpha {\;}_{i}}+{\bar{\lambda_{2}}}^{\alpha {\;}_{i}}+{\bar{\lambda_{3}}}^{\alpha {\;}_{i}}-3+\frac{1}{\beta_{i}}\left(J^{-\;\alpha_{i}\;\beta_{i}}-1\right)\right]$$

Where μ*_i_* are stiffness terms, α*_i_* specify the shape of the stress-strain curve and β*_i_* constants depend on Poisson’s ratios between principal stretch directions. The number of terms retained in the strain energy function was *N* = 2. Material properties given in the input to the FE model (see Table 1) were derived from a previous study via fitting of experimental data [33]. FE analysis included geometric nonlinearity to account for large deformations and buckling.

**Table 1:** Mechanical properties of the facesheet and core materials utilized in FE analyses.

Material	Constitutive Model	Elastic Constants [33]	Density [33]
Foam core	Hyperfoam (*N* = 2)	μ_1_ = 1.55 MPa, μ_2_ = 0.17 MPa, α_1_ = 13.8, α_2_ = 0.437	71.6 kg/m^3^
Facesheet	Elastic-plastic	*E* = 110 MPa, *ν* = 0.3, σ_yield_ = 7.4 MPa, *E*_plastic_ = 1.0 MPa	756.6 kg/m^3^

The out-of-plane displacements computed by ABAQUS were compared with the corresponding values measured experimentally with the two-projector moiré set-up. Once the FE model was validated against experimental measurements, it was parameterized with respect to: (i) the relative dimensions of the core and facesheets; and (ii) the waviness of the core-facesheet interfaces. All FE models were submitted to 10 mm end-shortening, the same value given to panels in the experimental tests.

**Figure 4:**
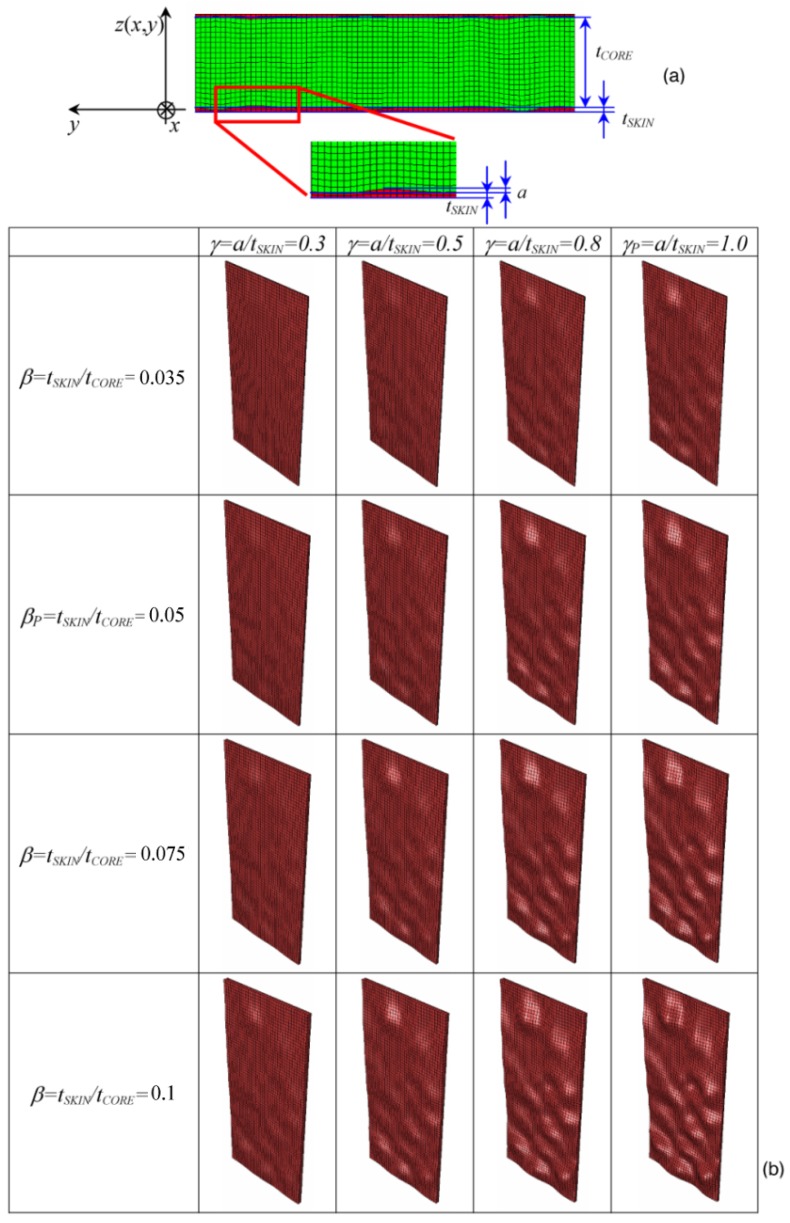
(**a**) Typical mesh considered in the parametric FE study on foam-based sandwich panels, including the nomenclature and reference system; and (**b**) facesheet meshes generated for different values of β and γ geometric parameters.

Let *t*_SKIN_ and *t*_CORE_ be the average thicknesses of facesheets and the core, respectively and *a*, the maximum deviation of the facesheet profile with respect to the plane defining the average thickness *t*_SKIN_ (see the detailed view of Figure 4a). The following geometrical parameters, β = *t*_SKIN_/*t*_CORE_ and γ = *a*/*t*_SKIN_, can be defined to describe the variation of model geometry. Specifically, for the panels tested in the experiments, we have β*_P_* = 0.05 and γ*_P_* = 1.0.

An *ad hoc* subroutine was written in MATLAB^®^ Version 7.0 (The MathWorks Inc., Austin, TX, USA) to parameterize the FE model. The routine changed the spatial coordinates of the nodes of facesheets and the core to realize the different combinations of β and γ illustrated in Figure 4b. The β ratio was hypothesized to assume four values: 0.035, 0.05, 0.075 and 0.1, respectively. The γ ratio also could take four values: 0.3, 0.5, 0.8 and 1.0, respectively. Therefore, 4 × 4 = 16 finite element analyses were performed in the sensitivity study. The total thickness of the panel *t*_P_* = t*_CORE _*+* 2*t*_SKIN_ = 42 mm was hypothesized to remain constant.

The chosen values of β and γ are very indicative. It is well known from the theory of sandwich structures that facesheets provide in-plane stiffness, while the core provides bending stiffness (see, for example, the classical textbook [34]). Therefore, by increasing β (*i.e.*, thicker facesheets) and γ (*i.e.*, a higher level of waviness in the inner facesheets), we expect to reduce the buckling strength of the panel, as the overall bending stiffness given by the core tends to decrease and the effect of geometric imperfections becomes more severe. Since FE analyses carried out in this study are nonlinear, because of the presence of geometric imperfections in the tested specimens, the sensitivity analysis on the effect of β and γ can give useful information on how the onset of buckling and the propagation of buckles through the panel change for different geometric configurations.

From a geometric point of view, changing the β ratio corresponds to a translation of the nodes in core-facesheet interfaces. Changing the γ ratio is equivalent to applying a transformation of dilation to the same nodes. Translation and dilation must be applied in two subsequent steps. In order to change the *t*_SKIN_/*t*_CORE_ ratio from the β*_P_* value determined for the panels tested in the experiments to a generic value β, the coordinates, z(*x,y*), of the interface nodes must be changed as follows (see Figure 4a):

(7) $$z_{\text{transl}}(x,y)=z_{\text{old}}(x,y)+t_{P}\cdot \left(\frac{\beta }{1+2\beta }-\frac{\beta_{P}}{1+2\beta_{P}}\right)$$

where *z*_old_(*x,y*) and z_transl_(*x,y*), respectively, are the nodal coordinates before and after translation. If *<z*_transl_(*x*,*y*)*>* is the average value of the coordinates, *z*(*x*,*y*), recomputed after node translation, the coordinates of the nodes of the “dilated” surface (*i.e.*, to pass from γ*_P_* to γ) can be written as:

(8) $$z_{\text{dilat}}(x,y)=<z_{\text{transl}}(x,y)>+\left(z_{\text{transl}}\left(x,y\right)\;-\text{​}<z_{\text{transl}}(x,y)>\right)\cdot \gamma$$

Equations (7) and (8) must be sequentially applied to transform the coordinates of the nodes at the core-facesheet interfaces. Consequently, the *t*_SKIN_/*t*_CORE_ and *a*/*t*_SKIN_ ratios change from their initial values, β*_P_* and γ*_P_*, to the generic values, β and γ. In the case with β = β*_P_*= 0.05 and γ = γ*_P_*= 1 (*i.e.*, tested specimens), from Equations (7) and (8) one finds: *z*_dilat_(*x,y*) *= z*_old_(*x,y*)*.* Coordinates *z*(*x*,*y*) of all other nodes that do not lie on the core-facesheet interfaces were properly changed, so as to preserve the regular shape of the elements.

## 3. Results and Discussion

**Figure 5:**
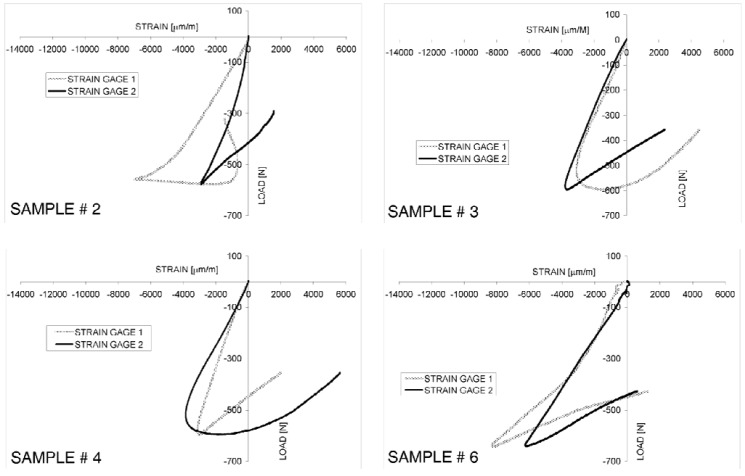
Typical strain-load curves obtained in the edgewise-compression tests of foam-based sandwich panels.

Strain-load curves recorded in the experiments always include a quasi-linear range corresponding to the Hookean behavior: for example, Figure 5 shows the strain-load curves obtained for samples 2, 3, 4 and 6. Beyond the peak load, the tendency line of the strain-load curve always presents a rather sudden change, which indicates the onset of buckling in the tested samples (see Figure 5a). The maximum load recorded in the experiments was on average 642 N, with a standard deviation of 63.3 N. The stiffness, determined as the ratio between the applied load and the end-shortening given to the panel in the Hookean regime, was 189.3 ± 15.2 N/mm for the population of tested specimens. The maximum apparent stress (*i.e.*, the ratio between the maximum load applied to the panel during the test and the nominal cross-sectional area of the specimen) was instead 0.18 ± 0.015 N/mm^2^.

FE analyses performed for the model reproducing the experimentally tested specimen (*i.e.*, with β = β*_P_*= 0.05 and γ = γ*_P_*= 1) showed that principal stresses in the loading direction for the core and the facesheets are on average in the ratio of 1 to 4.

Samples 3 and 6 showed a rather uneven initial strain response (however, this behavior was far more evident for the latter specimen). This could be due to a number of reasons. First, there may be some initial rigid body rotation of the specimen caused by imperfect clamping. Second, in spite of the care taken during the cutting operation, one facesheet of the sample could be slightly longer than the other (however, less than 100 ÷ 200 μm difference); this amplifies the effect of the inherent asymmetry of the foam core sandwich wall that included irregular facesheets with different profiles of the core/facesheet interfaces. Third, distribution of pores in the core and, hence, their closure rate may locally change for each specimen. However, it appears from Figure 5 that the load-strain response soon returned to regular for all specimens.

**Figure 6:**
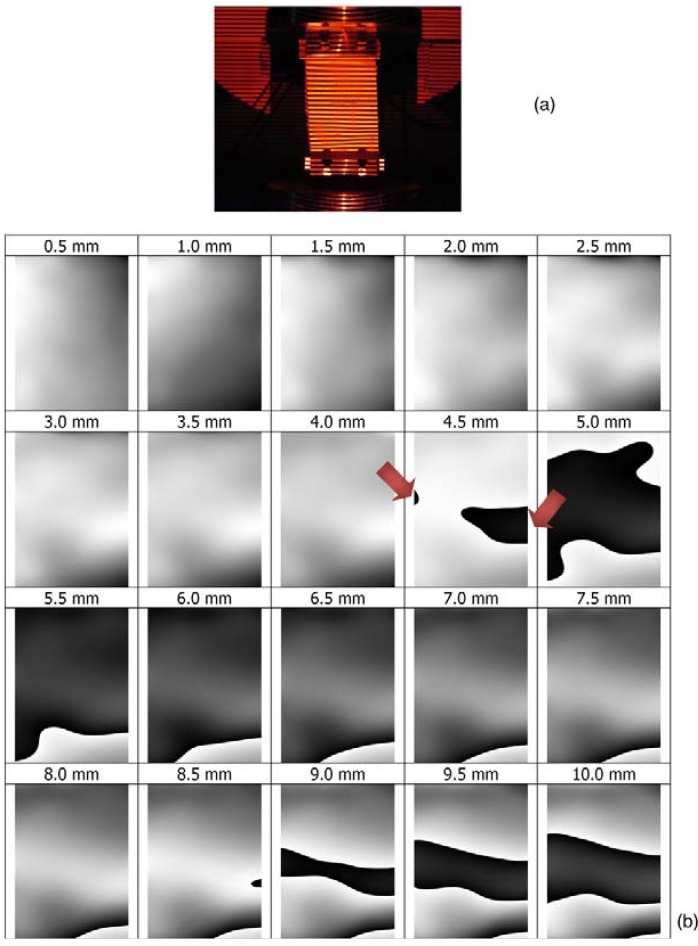

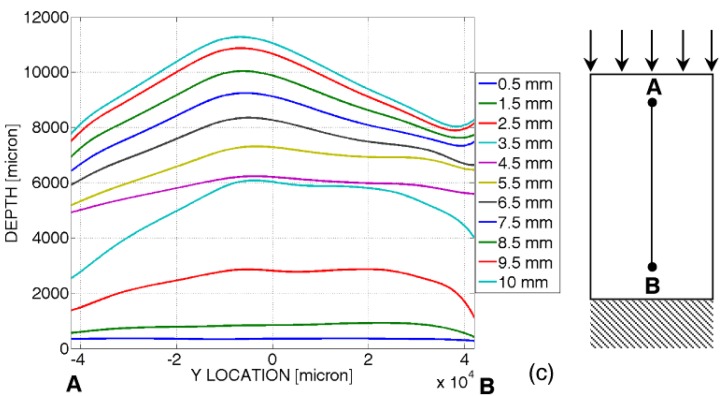
(**a**) Pattern of fringes projected by the bottom projector onto the loaded sample 2; (**b**) evolution of total phase difference, $\Delta \phi_{\text{TOT}}$, during the compression test; and (**c**) measured out-of-plane displacements along the AB control path for different levels of end-shortening given to the panel.

Moiré patterns were processed with the Holo Moiré Strain Analyzer™ (HMSA) Version 2.0 software (Sciammarella and Associates Ltd., Chicago, IL, USA), based on Fourier analysis [21,22]. The patterns of straight lines projected onto the undeformed specimen are modulated by the panel that deforms under the compression load (see, for example, Figure 6a, referring to sample 2). The detailed analysis of projection moiré measurements reveals that buckles originate in very localized regions near the edges of the panel and then propagate towards the center of the specimen (this is indicated by the red arrows in Figure 6b). It is worthy to note that the formation of a new fringe in the total phase map $\Delta \phi_{\text{TOT}}(x,y)$ (*i.e.*, the dark area that appears between 4.5 and 5 mm end-shortening)corresponds to increasing the value of out-of-plane displacement by one sensitivity Δ*S*.

By utilizing a full-field technique, like projection moiré, it is hence possible to monitor the evolution of the panel deformation along any control path. For example, Figure 6c plots the displacement distributions measured along the AB path corresponding to the specimen centerline in the longitudinal direction for different values of end-shortening given to the specimen.

The strain-load curves registered in the edgewise compression tests are consistent with the out-of-plane displacement maps found by projection moiré. However, only the optical technique could capture in real time the very localized onset of buckling that usually occurs where the imperfections of core-facesheet interfaces are more pronounced. For example, in the case of specimen 2, while projection moiré detected buckles to form already between four and 4.5 mm end-shortening (*i.e.*, less than 50% of the 10 mm end-shortening given to the panel), the sudden change in the strain-load curve trend was seen to occur at about 5 mm end-shortening, that is, only when buckles reached the position where the strain gage is glued to the facesheet. In other words, strain gages registered buckling onset with a delay with respect to its actual onset; such a delay corresponds to the time interval required by the buckles to propagate from the regions where they formed to the region where the strain gage is placed.

**Figure 7:**
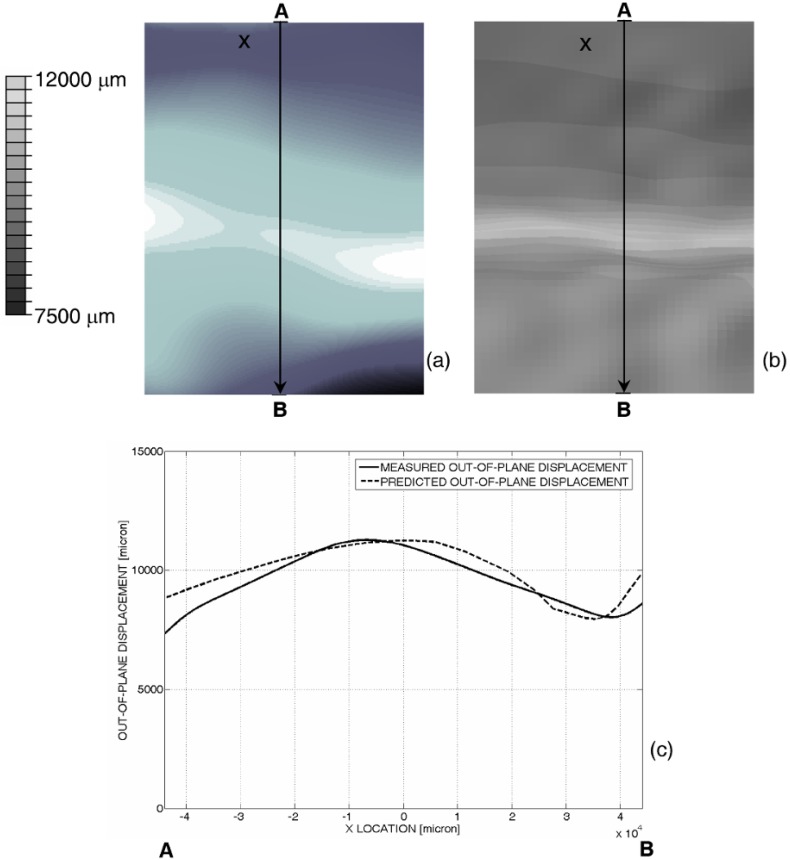
Comparison of out-of-plane displacement maps (**a**) measured with projection moiré; or (**b**) computed by ABAQUS for the 10 mm end-shortening given to the panel; and (**c**) displacement distributions compared along the AB control path.

Results of finite element analysis are consistent with experimental data. In fact, the out-of-plane displacement map computed by ABAQUS for the experimentally set 10 mm end-shortening (Figure 7b) reproduces the corresponding displacement map rather well measured with projection moiré (Figure 7a). Figure 7c shows that the wavelength of the buckling pattern computed by ABAQUS along the longitudinal control path AB is very close to that derived from moiré measurements.

The residual difference between experimental data and numerical results can be explained as follows. In the first place, the core was modeled as a continuum structure, while in reality, it is a porous structure. A very detailed reconstruction of the core geometry might have been achieved by performing micro-CT scanning. However, computation time may increase by orders of magnitude without improving results significantly [35,36,37,38]. Second, the present FE model takes into account only the local variations in facesheet thickness, but neglects all other manufacturing defects that may be included in the core and facesheets. Third, in spite of the high level of accuracy entailed by model definition, boundary and loading conditions still might not completely reproduce the actual conditions realized experimentally. Finally, mechanical properties implemented in the FE model for facesheet and core materials may be affected by some uncertainty.

**Figure 8:**
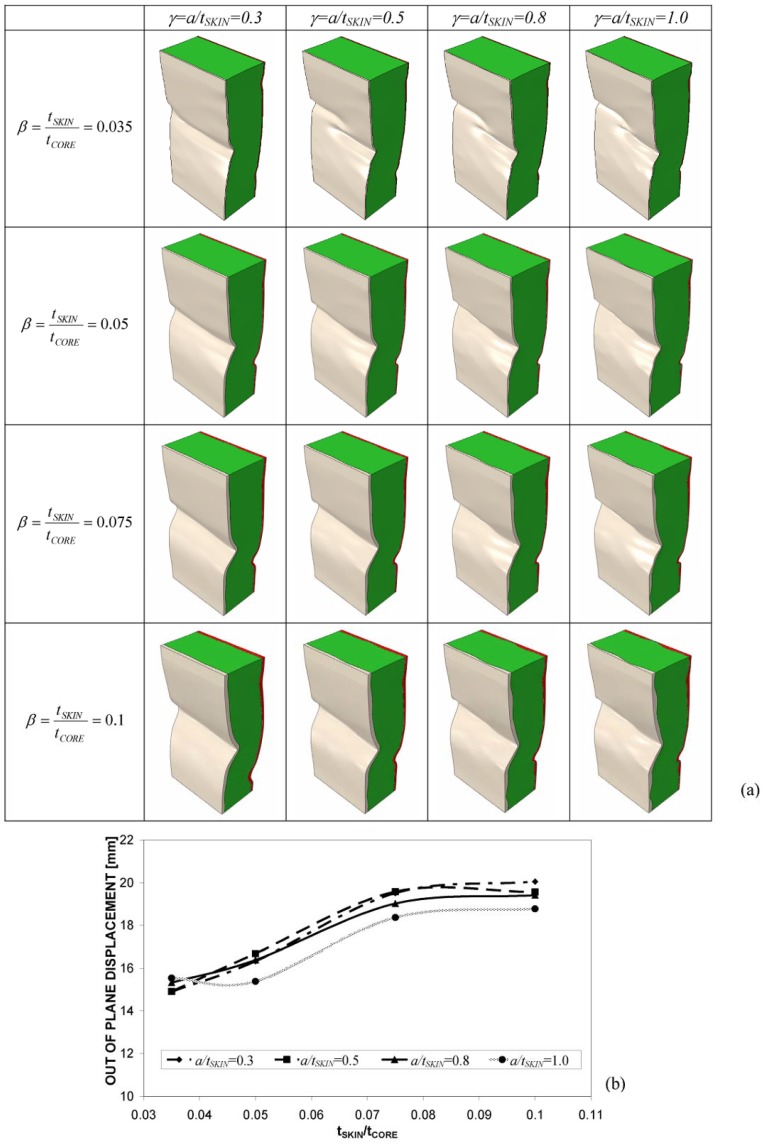
(**a**) Deformed shapes of the foam-based sandwich panel computed by parametric FE analysis for different values of β and γ (skins are colored in grey and red, while the core is colored in green; and (**b**) Variation of total out-of-plane displacement, *u*_TOT_, with respect to β for each value of γ considered in the parametric study.

In spite of the above mentioned limitations, the FE model reproduced the real behavior of the tested panels fairly well. Therefore, it is logical to carry out the sensitivity analysis on the effect of geometric parameters β and γ. Figure 8 presents the deformed shapes determined for the 16 panel configurations considered in the sensitivity study. Since the profiles of core-facesheet interfaces are different, the buckling mode of the panel is not symmetric; this is more evident for thicker facesheets.

It can be seen that the total out-of-plane displacement, *u*_TOT_, defined as the difference between the maximum positive and the minimum negative deflections experienced by the panel, increases with the ratio β between facesheet thickness and core thickness (Figure 8a). In general, the flexural stiffness of sandwich constructions is proportional to facesheet thickness and to the square of core thickness [34]. If the total thickness of the sandwich panel is kept constant (such as occurs in the present study), reducing core thickness may affect out-of-plane deformability more than increasing facesheet thickness, especially when there are imperfections at the facesheet/core interface that increase in magnitude for thicker facesheets and contribute to weakening the elastic foundation provided by the core. Consequently, the panel becomes more sensitive to buckling. In fact, for a given load level, nonlinear FE analyses reveal that buckles increase in size more rapidly. For example, for the value γ*_p_* = 1 corresponding to the real geometry of the panels tested in the experiments, the end-shortening yielding the experimentally measured deformation of 15 mm drops down from the 10 mm applied in the tests to only 8.85 mm as β increases from 0.05 (tested panels) to 0.1 (facesheets two times thicker than in the tested panels).

It can be seen from Figure 8 that the variation of the panel response in terms of total displacement is much less sensitive to the facesheet waviness parameter, γ, than to the facesheet-to-core thickness ratio, β. In fact, for a given value of β, the deformed shape of the panel does not change significantly, and the positions of deformed shape peaks and valleys defining the buckling mode wavelength stay almost the same regardless of the amplitude of facesheet imperfections quantified by the γ parameter. It should be noted that since the wavelength of core/facesheet interface imperfections (*i.e.*, the waviness periodicity) was much shorter than the wavelength of the buckling pattern, the structural response of the panel is affected to a limited extent by facesheet waviness.

The maximum stress developed in the panel is rather insensitive to the different combinations of β and γ, as it ranges from 13.3 to 14.6 MPa, with a tendency to increase slightly with β. The resultant load was much more sensitive to the skin to core thickness ratio and followed practically the same trend of variation with respect to β exhibited by the total out-of-plane displacement (see Figure 8b).

**Figure 9:**
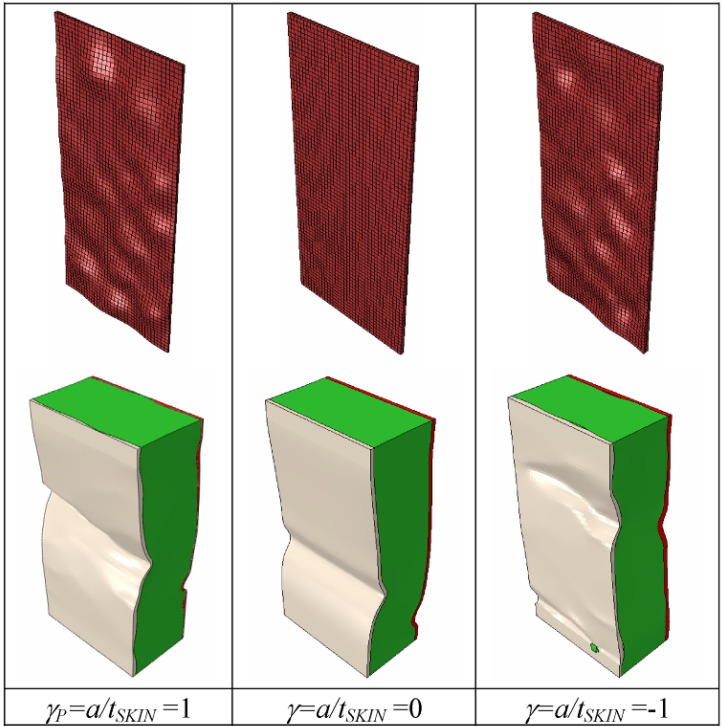
Deformed shapes computed by ABAQUS for γ = γ*_P_*= 1 (tested panels), γ= 0 (flat inner and outer facesheets) and γ = −1 (core-facesheet interface profile valleys are replaced by peaks and *vice versa*).

The observed behavior can be explained as follows. Buckling onset is very likely to occur in regions of the core-facesheet interface with larger imperfections, which grow as the compressive load given to the panel increases. However, stress concentrations in the core-facesheet interface depend on, for the most part, the curvature profile of the interface itself, which is on the relative positions of the interface profile peaks and valleys with respect to the line defining the average facesheet thickness. Since the dilation operation entailed by changing the γ parameter does not alter the nature and the relative positions of peaks/valleys (in simple words, the peaks remain peaks and the valleys remain valleys), it is reasonable to have a very similar buckled shape for a fixed end-shortening and a given value of the facesheet-to-core thickness ratio, β. In order to prove this statement, two additional FE analyses were run by setting γ = 0 (*i.e.*, flat skins of a thickness equal to the average thickness experimentally determined) and γ = 1 (*i.e.*, valleys are replaced by peaks and *vice versa*). The facesheet-to-core thickness ratio was set as β*_P_*, and the total end-shortening was assumed to be equal to 10 mm, thus reproducing the experimental conditions. As expected, buckled shapes were completely different (see Figure 9): this confirmed the dominant role of the shape of the core-facesheet interface profile.

Besides the issues of FE modeling and analysis, which, however, did not affect at all the validity of the present study, it might be argued that the two-projector set-up requires an accurate and time-consuming calibration procedure. In fact, projectors must be symmetric with respect to the optical axis of the CCD camera to realize the condition of projection from infinity. Furthermore, important parameters of the optical set-up (e.g., the depth of focus of the projectors, the intensity of the projected light, the pitch of the projected grating, *etc.*) must be exactly the same for both projectors. However, the calibration issue was greatly simplified in this research by designing a supporting frame that allows relative positions of projectors and sensor to be precisely controlled. Furthermore, the supporting system is flexible enough to allow for contouring objects with dimensions ranging from a few millimeters to some meters. With respect to simpler moiré set-ups employing only one projector, the two-projector set-up utilized in this study does not suffer from the inherent limitation on the size of the investigated object that, in the case of single projection, must be smaller than the size of the collimating lens that directs the structured light wavefront carrying the grating onto the specimen. Finally, the sensitivity of the two-projector set-up is twice as high as that of a single projector set-up, thus making it easier to achieve levels of accuracy up to 1/500 of the sensitivity of the measurement system.

## 4. Concluding Remarks

This paper analyzed the mechanical behavior of low density polyethylene foam core sandwich panels subject to edgewise compression. For that purpose, 10 specimens cut from a panel built via rotational molding were tested, and the strains developed in the facesheets were measured by means of strain-gauges, while out-of-plane deformation was monitored by a projection moiré set-up, including two projectors and one CCD camera. Compression tests were then simulated by a nonlinear FE model accounting for the real shape of the facesheet-core interfaces, material nonlinearity and large deformations. Once the FE model was validated against experimental data, parametric FE analyses were carried out to assess to what extent the mechanical response of the panel is sensitive to the facesheet-to-core thickness ratio and the waviness of the core-facesheet interfaces.

It was found that projection moiré can monitor the complete evolution of buckling from the onset in very localized regions near panel edges to the propagation throughout the panel. Conversely, strain gages exhibited some delay in capturing buckling phenomena. Calibration issues usually entailed by moiré measurements were solved in this study by designing a supporting frame that allows the relative positions of projectors and sensor to be precisely controlled.

FE computations were in good agreement with experimental evidence. The sensitivity analysis indicated that the core must be thick enough to prevent buckling, which in low density polyethylene foam-based sandwich panels built by rotational molding is a progressive phenomenon, due to the presence of geometric imperfections in the core-facesheet interfaces. However, for a given facesheet-to-core thickness ratio, the local distribution of core-facesheet interface waviness seems to be more important than the amplitude of interface imperfections.
